# Crizotinib and Doxorubicin Cooperatively Reduces Drug Resistance by Mitigating MDR1 to Increase Hepatocellular Carcinoma Cells Death

**DOI:** 10.3389/fonc.2021.650052

**Published:** 2021-05-18

**Authors:** Ming Shao, Run Shi, Zhen-Xing Gao, Shan-Shan Gao, Jing-Feng Li, Huan Li, Shu-Zhong Cui, Wei-Min Hu, Tian-Yun Chen, Gui-Ru Wu, Jie Zhang, Jiang Xu, Man-Sun Sy, Chaoyang Li

**Affiliations:** ^1^ State Key Laboratory of Virology, Wuhan Institute of Virology, Chinese Academy of Sciences, Wuhan, China; ^2^ University of Chinese Academy of Sciences, Beijing, China; ^3^ State Key Laboratory of Respiratory Disease, Affiliated Cancer Hospital and Institute of Guangzhou Medical University, Guangzhou, China; ^4^ Abdominal Surgery, Affiliated Cancer Hospital and Institute of Guangzhou Medical University, Guangzhou, China; ^5^ Department of Stomatology, First Affiliated Hospital of Nanchang University, Nanchang, China; ^6^ Department of Stomatology, First Affiliated Hospital, School of Medicine, Shihezi University, Shihezi, China; ^7^ Department of Pathology, School of Medicine, Case Western Reserve University, Cleveland, OH, United States

**Keywords:** PERK, multi-drug resistant protein 1, hepatocellular carcinoma, ER stress, autophagic cell death

## Abstract

As the sixth most lethal cancers worldwide, hepatocellular carcinoma (HCC) has been treated with doxorubicin (Dox) for decades. However, chemotherapy resistance, especially for Dox is an even more prominent problem due to its high cardiotoxicity. To find a regimen to reduce Dox resistance, and identify the mechanisms behind it, we tried to identify combination of drugs that can overcome drug resistance by screening tyrosine kinase inhibitor(s) with Dox with various HCC cell lines *in vitro* and *in vivo*. We report here that combination of Crizo and Dox has a synergistic effect on inducing HCC cell death. Accordingly, Crizo plus Dox increases Dox accumulation in nucleus 3-16 times compared to Dox only; HCC cell death enhanced at least 50% *in vitro* and tumor weights reduced ranging from 35 to 65%. Combining these two drugs reduces multiple drug resistance 1 (MDR1) protein as a result of activation of protein kinase RNA-like endoplasmic reticulum kinase (PERK), which phosphorylates eIF2α, leading to protein translational repression. Additionally, PERK stimulation activates C-Jun terminal kinase (JNK), resulting in accumulation of unfused autophagosome to enhance autophagic cell death *via* Poly-ADP-ribosyltransferase (PARP-1) cleavage. When the activity of PERK or JNK is blocked, unfused autophagosome is diminished, cleaved PARP-1 is reduced, and cell death is abated. Therefore, Crizo plus Dox sensitize HCC drug resistance by engaging PERK-p- eIF2α-MDR1, and kill HCC cells by engaging PERK-JNK- autophagic cell death pathways. These newly discovered mechanisms of Crizo plus Dox not only provide a potential treatment for HCC but also point to an approach to overcome MDR1 related drug resistance in other cancers.

## Introduction

Hepatocellular carcinoma (HCC) is the sixth most malignant cancers worldwide responsible for 745,000 deaths in 2012 (1). Furthermore, the incidence and death rates of HCC have been rising (2). Due to oblivious early symptoms, many patients are not diagnosed at the early stage when curative surgical intervention or transplantation are options. Thus, the overall 5-year survival rate of patients with HCC is less than 15% (3, 4).

Doxorubicin (Dox) is a DNA intercalation agent and an inhibitor of topoisomerase II (5). It is thought that Dox induces cell death by causing genotoxic effects, eventually leading to cell death (6). Since 1974, Dox has been used as a first line chemotherapy drug to treat a plethora of malignancies, including HCC (7, 8). However, drug resistance, represented by the up-regulation of Dox efflux pump multiple drug resistance 1 (MDR1) limits the efficacy of Dox (9, 10). In addition, high toxicity, especially cardiotoxicity, and low response rate imposes an even more prominent limit in the use of Dox monotherapy (11). To overcome these problems, combinatory regimens of Dox with cisplatin, interferon or fluorouracil or nanocarrier targeting tumor have been explored to treat advanced HCC (12, 13). Unfortunately, these approaches have not produced obvious advantage over Dox monotherapy (14).

Aberrant signal activation has been observed in HCC (15–20). To target these aberrant signaling cascades, two non-selective kinase inhibitors have been approved for treating advanced HCC: Sorafenib and Regorafenib. Still, both drugs extend the median overall survival of patients with advanced HCC by less than 3 months (21–23). Therefore, new targets or reformulation of existing drugs are urgently needed.

For this purpose, we screened HCC cell lines with some non-receptor/receptor tyrosine kinase inhibitors (TKIs) in preliminary experiments. We find that Crizo is relatively more effective in killing HCC cells. Thus, we use Crizo plus Dox to investigate whether the combination could kill cancer cells more efficiently. We find that Dox and Crizo show synergistic effects in inducing HCC cell death compared to Dox or Crizo alone. This synergy is enhanced by the activation of the ER stress sensor, protein kinase RNA-like endoplasmic reticulum kinase (PERK), which then engages autophagic cell death pathway resulting in enhanced cell death. Finally, we show that Crizo significantly promotes Dox’s effect in inhibiting HCC cell growth *in vivo* in xenograft models. The significances of these findings are discussed.

## Materials and Methods

### Cell Culture and Agents

Certified human hepatocellular carcinoma cell lines BEL7402 (7402), HLF, SK-hep1 and HepG2 and non-HCC cell L02 free of mycoplasma contamination were purchased from China Center for Type Culture Collection. All cell lines were cultured in high glucose Dulbecco’s modified Eagle’s medium (DMEM, Gibco, USA) containing 10% fetal bovine serum (FBS, Hyclone) and 1% penicillin-streptomycin (Biological Industries, BI). The cells were maintained at 37°C in a 95% humidified 5% CO2 atmosphere. Doxorubicin (Dox), crizotinib (Crizo), 3-MA, MG132, GSK2606414 (GSK), SB203580 (SB), INO-1001 (INO), chloroquine (CQ), AG490, LY294002, BGJ398, BMS754807, ZM306416, Ki8751, SAR131675, Axininib, and Murbritinib were purchased from Selleck (China, Shanghai). Caspase-3 inhibitor II (C3-I) was purchased from Millipore, USA. These following antibodies (Abs) were purchased from cell signaling technologies: PARP1 (9542#), full length caspase-3 (9668#), cleaved caspase3 (9661#), p-JNK (4668#), MDR1 (13342#), p-EIF2a (3398#), JNK (9252#) and EIF2a (5324#). LC3B (sc-398822) and p62 (sc-28359) were purchased from Santa Cruz Biotechnology. Ab specific to β-actin was purchased from SUNGENE BIOTECH (Tianjin, China). HRP conjugated goat anti-rabbit or anti-mouse secondary Abs were purchased from ABclonal (Wuhan, China). MTS (3-(4,5-dimethylthiazol-2-yl)-5-(3-carboxymethoxyphenyl)-2-(4-sulfophenyl)-2H-tetrazolium) was purchased from Promega; DAPI was purchased from ThermoFisher Scientific. Apoptosis assay kit was purchased from Beyotime, China. Alexa Fluor conjugated secondary Abs were purchased from Invitrogen. LC3-GFP or LC3-GFP-mcherry was kindly provided by Professor Ming-Zhou Chen at Wuhan University, China.

### Cell Viability Assay

Cytotoxicity of Dox, Crizo and other TKIs on the viability of HCC cell lines was assessed by MTS assay. Briefly, 5000 cells/well of each cell line were seeded as triplicates in 96 well plates eight hours before drug treatment. Dox (0.5 μM), or Crizo (5 μM), or Dox (0.5 μM) plus Crizo (5 μM) were diluted in culture medium at indicated concentrations. The culture medium without drug was replaced by culture medium in the presence of drugs or vehicle for 24 hours. At the end of the experiment, 20 μl MTS were added to each well and incubated with the cells in the 37°C incubator for 2 additional hours (hs). The plate was then detected at 492 nm wavelength optical density (OD).

To assay the effect of specific signaling cascade on HCC cells treated with Dox plus Crizo, MTS assay was performed as above for different treatments. vehicle, GSK (0.5 μM), INO (1.0 μM), SB (4.0 μM), C3-I (0.5 μM) or CQ (50 μM) was added into HCC cells incubated with fresh medium containing 0.85 μM Dox+5 μM Crizo in 96-well plates for 48 hs. At the end of the experiment, 20 μl MTS were added to each well and incubated with the cells in the 37°C incubator for 2 additional hs. The plate was then detected at 492 nm OD. Relative cell survival (%) = (OD value of the experimental group normalized to OD value of the vehicle control group) ×100%. Relative cell proliferation rate= (OD value of the group at the indicated time point normalized to OD value of the same group at time 0).

### Long-Time Proliferation Assay (Clonal Formation Assay)

5000 cells per well were seeded in 6-well plates. After culture for 12 hs, vehicle, 20 nM Dox, 0.6 μM Crizo and 20 nM Dox+0.6 μM Crizo diluted in fresh medium were used to replace the existing medium every three days, for a period of 10 days incubation. At the end of the experiment, the medium was discarded and the cells were rinsed twice with 1X phosphate buffered saline (PBS). Then cells were fixed with 4% paraformaldehyde (PFA) for 20 minutes (min), and stained with 0.1% crystal violet for 30 min. The plate was washed with clean water until no residual dye. At last, the plate was air dried and pictures were taken.

### Determination of Half of Maximal Inhibitory Concentration (IC50) and Combination Index (CI)

7402, HLF and HepG2 cells (5000 cells/well) were plated in 96-well plates and treated with increasing concentrations of Dox or Crizo for 48 hs. In order to determine the combination effect of Dox and Crizo on HCC cells, cells were treated with increasing concentrations of Dox and Crizo in a non-fixed ratio of IC50 of the two agents for 48 hs. Cell viability was detected using MTS as above. IC50 was calculated by CompuSyn software. Synergistic effect was calculated according to Chou et al. (24). A CI value less than1 indicates a synergistic effect of the combination of drugs.

### Apoptosis Assays: Annexin V/Propidium Iodide Staining

Drug-induced cell apoptosis was determined by flow cytometry using an annexin V-FITC/propidium iodide (PI) apoptosis detection kit according to the manufacturer’s instructions. Briefly, cells were treated with vehicle control, Dox (0.85 μM), Crizo and Dox (0.85 μM) + Crizo (5 μM) for 24 hs. The cells were then trypsinized, rinsed twice with ice-cold PBS, and centrifuged at 1,800X g. Resuspended cells (1x10^5^ cell/ml) were mixed with 195 μl Annexin V-FITC binding buffer, and then stained with 5 μl Annexin V-FITC and 10 μl PI at room temperature (RT) for 15 mins in dark, and then subjected to flow cytometry analysis with a FACSCanto flow cytometer (BD Biosciences). 10,000 cells per treatment were acquired for each sample. Data analysis was performed using FlowJo software, version 7.5.5 (Tree Star Inc., Ashland, OR).

### Immunoblotting

After 24 hs treatment with vehicle control, Dox (0.85 μM), Crizo (5 μM) or Dox (0.85 μM)+Crizo (5 μM), the cells were rinsed three times with ice-cold PBS, and solubilized in lysis buffer (20 mM Tris-HCl (pH 7.5), 150 mM NaCl, 1 mM EDTA, 1 mM EGTA, 1% Triton X-100, 2.5 mM sodium pyrophosphate, 1 mM β-glycerol phosphate, 1 mM Na3VO4, 1mM phenylmethylsulfonyl fluoride (PMSF) and a protease inhibitor cocktail). Western blot analysis was performed as described previously (25). Briefly, protein concentration was determined by Bio-Rad Protein Assay kit (Bio-Rad). The cell lysates containing the same amount of total proteins were then mixed with 4 × sodium dodecyl sulfate (SDS) loading buffer, and boiled at 100°C for 10 mins. Equal amount of denatured protein (50 μg) from each sample was separated by the 10% SDS-polyacrylamide gel electrophoresis (PAGE). The separated protein was transferred to a 0.4 μm nitrocellulose membrane (Merck Millipore, USA) and blocked in 5% non-fat milk in TBST (Tris buffered saline (TBS) with 0.1% Tween-20). The separated proteins were probed with corresponding primary Abs as indicated. Bound primary Abs were further probed with HRP conjugated secondary Abs. The quantification of indicated proteins was based on densitometry using the Image J software (NIH).

### Inhibitor of Protein Degradation Assays

To investigate if MDR1 protein was degraded due to Dox (0.85 μM) + Crizo (5 μM) treatment, vehicle, 10 μM MG132, 50 μM CQ or 4 mM 3-MA were diluted with fresh culture medium containing 0.85 μM Dox and 5 μM Crizo and then added to 6-well plate containing HCC cells for 4 additional hs. To assess the function of the inhibitors, cells were seeded in a 12-well plate overnight, fresh medium containing 0.85 μM Dox + 5 μM Crizo was used to treat cells for 18 hs. After replacing the culture medium, inhibitors (GSK, SB, or INO) at indicated concentration or vehicle were added to fresh culture medium having 0.85 μM Dox + 5 μM Crizo and incubated with HCC cells for an additional 2 hs. After that, cell lysates were made and protein was quantified as above. Separated proteins were subjected to immunoblotting with specified antibodies.

### Dox Accumulation Assay

Cells (5x10^4^) were subcultured on the 20 mm glass bottomed dishes (NEST, China) overnight. Cells were then maintained in culture medium supplemented with drugs (0.85 μM Dox, 5 μM Crizo alone or in combination) for 24 hs. After rinse with PBS three times, cells were immediately fixed with 4% PFA for 20 mins at RT. After additional rinse three times with PBS, the cells were photographed with an Olympus fluorescence microscopy (UltraView Vox confocal microscope, Perkin Elmer). Dox in each indicated sample was quantified based on densitometry using the Image J software (NIH).

### Immunofluorescence Staining

To detect MDR1 or LC3-II, cells were cultured in the 20 mm glass bottomed dishes overnight for immunofluorescence microscopy observation. Cells treated with 0.85 μM Dox or/and 5 μM Crizo were fixed in 4%PFA for 15 mins at 25°C. After blocking with 10% normal goat serum plus 1% bovine serum albumin (BSA) in PBS containing tween 20 (PBST) at RT for 1 h, the cells were then incubated with corresponding primary Abs in blocking buffer overnight at 4°C. Bound Ab was then probed with Alexa Fluor conjugated secondary Abs for 1 hour at 25°C in dark. The nuclei were counterstained with DAPI (500 ng/ml) for 5 mins. After mounting with anti-fade fluorescence medium (Beyotime; P0126), the cells were imaged by a fluorescence microscopy. Fluorescence intensity was analyzed by the Image J software (NIH).

To investigate if autophagosome formation was influenced by PERK-JNK signaling cascade, cells were treated with 0.85 μM Dox plus 5 μM Crizo in the presence of vehicle, or 1 μM GSK, or 5μM SB for an additional 12 hs. The treated cells were then fixed with 4% PFA for 15 minutes at RT. LC3 A/B (#12741, CST) immunofluorescence staining was performed as above. The autophagosome flux is represented by the intensity of the LC3A/B staining. Immunofluorescence intensity of HCC cells treated with vehicle is arbitrarily defined as 1. Flux of treatment is calculated as: immunofluorescence intensity of cells treated by inhibitor/immunofluorescence intensity of HCC cells treated with vehicle. The intensity is determined by the Image J software (NIH).

To explore whether the two drugs combination treatment affects autophagosome accumulation and inhibits autolysosome formation, mCherry-GFP-LC3 plasmid (2 μg) was transfected into 7402 and HLF cells in a six-well plate, respectively. After 12 hs, transfected cells were reseeded in glass bottomed confocal dishes for an additional 24 hs. Full medium containing vehicle, 0.85 μM Dox or/and 5 μM Crizo were added to replace the normal medium for an extra 6 hs. The cells were then fixed and observed through a Nikon two-photon super resolution fluorescence microscope (Nikon A1 MP STORM). EGFP and mCherry positive indicate autophagosome, only mCherry positive represents autolysosome which are the fusion product of autophagosome and lysosome.

### Tumor Xenograft In Vivo

Six to eight-week-old male BALB/c nude mice were purchased from Beijing Vital River Laboratory Animal Technology Company for *in vivo* xenograft experiments. The animals were kept in SPF-II conditions and the animal experiment protocol (WIVA28201703) was approved by the animal care and use committee of Wuhan Institute of Virology, Chinese Academy of Sciences (Wuhan, China). Briefly, 1x10^7^ HepG2 or 7402 cells were suspended in 100 μl PBS, respectively and then inoculated subcutaneously into the right-back of each mouse. When the average tumor volume reached 150-200 mm^3^, Mice were randomly divided into 4 groups according to different treatments: for HepG2 cell, vehicle control group (0.5% sodium carboxymethyl cellulose by oral gavage and PBS for intraperitoneal (IP) injection), Dox group (5 mg/kg every two days IP), Crizo group (50 mg/kg/day by oral gavage), and Dox+Crizo group (5 mg/kg dox every two days IP and 50 mg/kg/day by oral gavage); for 7402 cell, the Dox concentration was 2.5 mg/kg, other conditions were the same as HepG2. The body weight and tumor volume of each mouse were measured every other day as indicated in the figure. The tumor volume (V) was estimated according to the following formula: V=0.5 x length x width x width. After two weeks treatment, the mice were euthanized, and the bearing tumors of each group were removed and weighed. The tumors from different treatment group were lined together and photographed. Note: Dox was dissolved in PBS for IP injection; Crizo was grounded as fine powder in a mortar and pestle, suspended in 0.5% sodium carboxymethyl cellulose.

### Statistical Analyses

The data are presented as means ± SEM of indicated experiments. One-way ANOVA was performed to analyze differences among the groups. Other statistical analyses were conducted by the two tailed Student’s t-test. *p<0.05 and **p<0.01, ***p<0.001were considered to be a significant difference.

## Results

### Crizo and Dox Act Synergistically to Enhance Human HCC Cell Death

**Figure 1:**
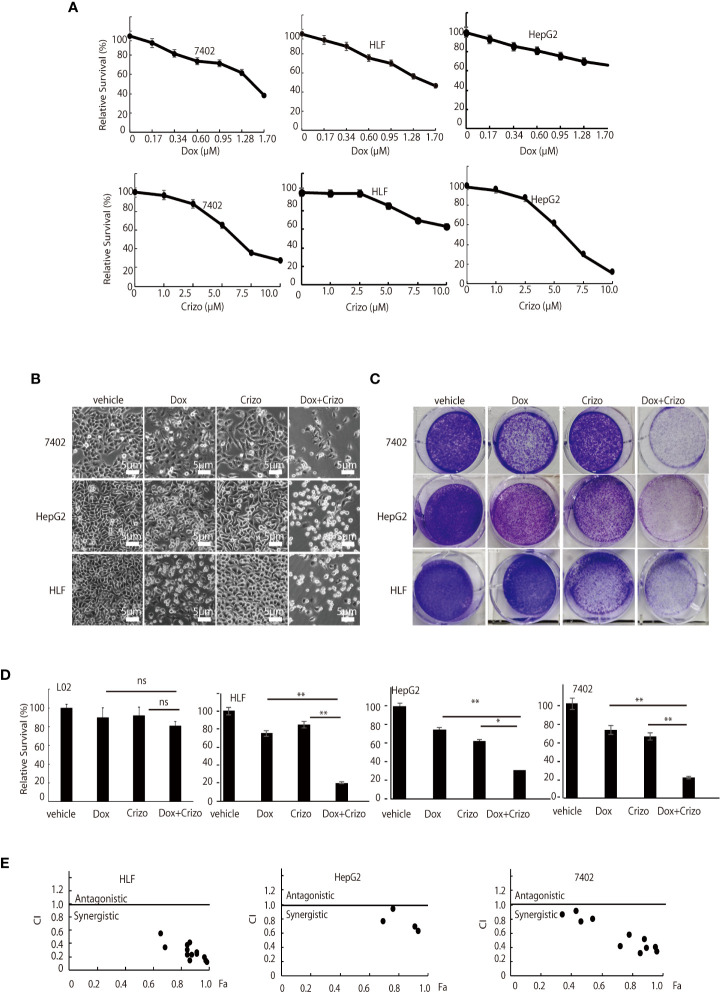
Dox and Crizo treatment synergistically induces hepatocellular carcinoma cell death. **(A)** HFL was relatively resistant to Crizo based on MTS assays under the tested conditions. Cells were treated with different concentrations of Dox or Crizo for 24 hs and quantified by MTS. Number of cells with vehicle treatment was arbitrarily defined as 100%, percentage of survival was the ratio of number of cells treated with Dox or Crizo to number of cells with vehicle treatment under the same condition. **(B)** Relative survival of 7402, HepG2, and HLF cells was monitored by bright field observation at 24 hours after the cells were treated with Dox (0.5 μM), or Crizo (5 μM), or Dox (0.5 μM) plus Crizo (5 μM). The number of cells at the time 0 of drug treatment was arbitrarily defined as 1. Scale bar:5 μm. Relative cell survival was the number of cells at indicated time treated with indicated drugs normalized to the number of cells at the time 0 of drug treatment. **(C)** Clonal formation of different HCC cells showed that significantly fewer cells survived Dox plus Crizo treatment. Cells were treated with vehicle, Dox, Crizo, or Dox plus Crizo as indicated. **(D)** MTS Quantification of four different experiments with L02, HLF, HepG2, and 7402 showed that compared to Dox or Crizo treatment separately, Crizo plus Dox induced significantly more cells death in cancer cells but not in L02. HCC cells were treated with vehicle, Dox (0.5 μM), Crizo (5 μM), or Crizo (5 μM) plus Dox (0.5 μM) for 24 hours and survived cells were monitored by MTS assays. Percentage cell survival was calculated as in 1 **(A)**. **(E)** To calculate combination index (CI) (CompuSyn software), combinations of different concentrations of Crizo, Dox, or Crizo plus Dox were applied to treat HCC cells for 48 hs and cell survival was monitored by MTS, it turned out that Crizo plus Dox showed synergistic effects in killing HCC cells under some combinations of Dox and Crizo even for HLF and HepG2 cells. Fa: fractions of the system affected. The experiments were repeated at least three times with similar results. *P < 0.05; **P < 0.01. ns, statistically no significant difference.

First, we investigated if Dox could kill human HCC cell lines maintained in our lab. We find that indeed Dox is able to kill the tested cells although HepG2 cells are relatively more resistant to Dox (**Figure 1A**, top panel). We then screened the effect of different TKIs available on HCC cell lines in a preliminary experiment. It turns out that Crizo is the relatively effective TKI in inducing cell death of HepG2 and 7402 (**Figure 1A**, bottom. Results for other TKIs were not shown). On the other hand, HLF are relatively more resistant to Crizo (**Figure 1A**, bottom panel). Thus, each cell line has its unique response to Crizo and Dox.

Since Dox and Crizo are capable of killing HCC cells, we investigate whether Crizo and Dox act synergistically in promoting HCC cell death. We cultured the HCC cells with Crizo (5 μM), Dox (0.5 μM), or Crizo (5 μM) plus Dox (0.5 μM). Crizo and Dox treatment increases at least 50% more cell death compared to Dox or Crizo treatment only. The effect is valid even for HepG2 and HLF cells, which are relatively more resistant to Dox or Crizo (**Figure 1B**). This observation is confirmed in colony forming assays (**Figure 1C**). We then investigate the effect of Dox and Crizo on HLF, 7402, and HepG2 HCC cells and on a non-HCC L02 cells, we found that Dox plus Crizo did not have obvious effects on L02 but showed a synergistic effect on HCC cells compared to Dox or Crizo treatment only (**Figure 1D**). In contrast, we did not observe synergism between Dox and other tested TKIs on HCC cells (Results not shown). Based on these observations, we then investigate the synergistic effects of Crizo plus Dox by varying the concentrations of each drug in the combination and calculated the Combination Index (CI) for each combination to determine whether the interaction is synergistic or additive. A CI value less than 1 is considered to be synergistic (24). Indeed, Crizo plus Dox show synergistic effect in all tested cell lines in different combinations (**Figure 1E**). The combinations showing synergistic effects of the drugs to four HCC cell lines are shown in **Supplemental Table 1**.

### Crizo Significantly Promotes Dox’s Effect on Human HCC Cell Death by Inducing PARP-1 Cleavage

**Figure 2:**
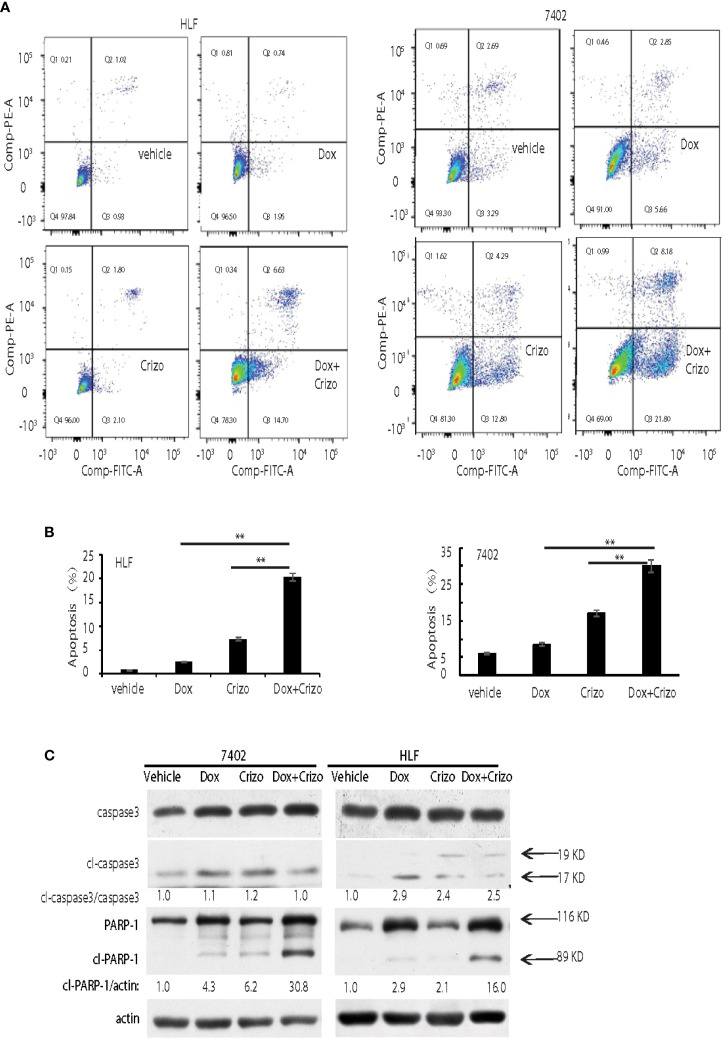
Crizo plus Dox show a synergistic effect in killing cancer cells by inducing PARP-1 cleavage. **(A)** Crizo (5 μM) plus Dox (0.85 μM) induced significantly more cell apoptosis compared to vehicle, Dox (0.85 μM), or Crizo (5 μM) only. Flow cytometry assays of HLF and 7402 cells showed that Crizo plus Dox treatment on these cells activated obviously more early and later apoptosis (Q3 and Q2) when compared to HCC cells treated only with vehicle, Dox, or Crizo. **(B)** Statistical analysis of three different experiments showed that Crizo plus Dox induced significantly more apoptosis than Dox or Crizo only on HCC cells. Percentage of apoptosis was defined as the combination of early and later apoptosis (Q2+Q3) (Filled in bar graph represented the mean and standard deviation). **(C)** Crizo plus Dox induced more cleaved PARP-1 compared to vehicle, Dox, or Crizo only when treating HCC cells. Immunoblotting of cell lysates from 7402, HLF were treated with vehicle, Dox (0.85 μM), Crizo (5 μM), or Dox (0.85 μM) plus Crizo (5 μM) with PARP-1 and caspase-3 specific antibodies showed more cleaved PARP-1 (cl-PARP-1) from cell lysates treated with Crizo plus Dox. In contrast, active caspase-3 (cl-caspase-3) was not significantly increased by Dox plus Crizo treatment. Cell treatment was performed the same as in **Figure 1**. Relative protein levels were determined with IMAGE J as specified. The experiments were repeated at least three times with similar results. **P < 0.01.

In mammalian cells, different pathways control the cell death programs, including apoptotic, autophagic and necrotic cell deaths (26). We then investigated which pathway is affected by Crizo and Dox in inducing HCC cell death. We cultured two HCC cell lines with Dox (0.85 μM), Crizo (5 μM), or Crizo (5 μM) plus Dox (0.85 μM), and quantified apoptosis by flow cytometry. We use these concentrations of Crizo and Dox in all subsequent experiments. Again, Crizo plus Dox activate significantly more apoptotic cell death in treated cell lines. A representative flow cytometry result is shown in **Figure 2A**. A summary of results from three independent experiments is shown in **Figure 2B**.

Apoptotic cell death involves the activation of caspase. One of the best characterized caspases is the executioner caspase, caspase 3 (26). Thus, we determine whether Crizo plus Dox activates caspase 3. It appears that Crizo or Dox by itself is able to slightly increase cleaved caspase 3, but the synergistic effect of Crizo plus Dox is not observed (**Figure 2C**). Thus, the synergistic effect of Crizo plus Dox in inducing HCC cell death is unlikely to be due to the activation of caspase 3.

Dox has been reported to induce autophagic death in 3T3 cells by activating Poly-ADP-ribosyltransferase (PARP-1) without involving caspases (27). Next, we investigated whether PARP-1 is involved in Crizo plus Dox induced cell death. HCC cell lines were treated with vehicle, Dox, Crizo, or Crizo plus Dox. Cell lysates were then prepared and immunoblotted. Much more cleaved PARP-1 as indicated by the presence of a smaller 89 kDa fragment is detected in the lysates from cells treated with Crizo plus Dox compared to all the other controls (**Figure 2C**).

### Crizo Plus Dox Significantly Increase Dox Accumulation in the Nucleus of HCC Cells by Modulating the Expression of MDR-1

**Figure 3:**
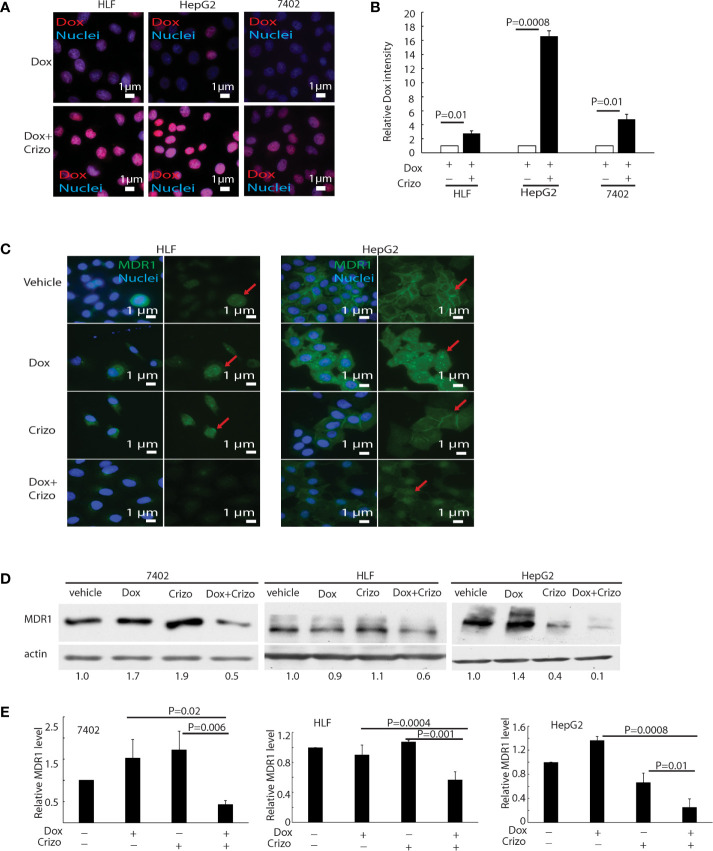
Crizo plus Dox induce Dox nuclei accumulation *via* reducing MDR1 protein levels. **(A)** Crizo (5 μM) plus Dox (0.85 μM) treatment of HCC cells resulted in more Dox (red) accumulation in the nuclei (blue) compared to Dox (0.85 μM) treatment only. Immunofluorescence pictures were taken after cells were treated for 24 hs. **(B)** Summary of three experiments showed that Dox plus Crizo resulted in significant more nuclei Dox accumulation. Open and filled in bar graphs represented the mean and standard deviation. **(C)** Immunofluorescence staining of MDR1 with specific antibody revealed that Crizo (5 μM) plus Dox (0.85 μM) treatment mitigated MDR1 expression than vehicle, Dox (0.85 μM), or Crizo (5 μM) only treatment. Cells were treated for 24 hs and then fixed for immunofluorescence staining. In HepG2 cells, Dox treatment seems changed the distribution of MDR1. **(D)** Significantly less MDR1 was detected from cell lysates made from HCC cells treated with Crizo (5 μM) plus Dox (0.85 μM) than from cells treated with vehicle, Dox (0.85 μM), or Crizo (5 μM) only. **(E)** Statistical analysis of three experiments showed that Dox plus Crizo resulted in significantly less MDR1 in HCC cells. Filled in bar graph represented the mean and standard deviation. Cells were treated for 24 hs before cell lysates were made and immunoblotted with antibodies specific to MDR1 or actin. Nuclei were counterstained by DAPI. Relative immunofluorescence intensity or protein levels were determined with IMAGE J as specified. The experiments were repeated three times with similar results. P values were indicated.

Dox must be able to enter and stay inside the nucleus to mediate its cytotoxic effect. Therefore, we investigated whether the synergistic effect of Crizo plus Dox is due to an increase in the accumulation of Dox in the nucleus of treated HCC cells. Only background signals of Dox are seen in the nucleus of Dox treated HCC cells (**Figure 3A**, top panels). However, in the presence of Crizo plus Dox, significantly more Dox signals are detected in the nucleus, ranging from 3 - 16 times dependent upon cell line (**Figure 3A**, bottom panels). Quantification of results from three independent experiments is shown in **Figure 3B**. Therefore, Crizo appears to promote the accumulation of Dox in the nucleus.

Accumulation of Dox in the nucleus suggests that either the influx of Dox is enhanced or the efflux of Dox is reduced or both. We thus detected if proteins involved in drug influx or efflux were affected by Dox plus Crizo treatment. We find that the expression of MDR1, a drug efflux protein (28, 29), is altered when HCC cells were treated with Crizo plus Dox. Immunofluorescence staining and confocal imaging reveal that the level of MDR1 is greatly reduced when HCC cells are treated with Crizo plus Dox as compared to control cells (**Figure 3C**). In addition to cell membrane staining, there is some nuclear staining of MDR1 in HepG2 cells (**Figure 3C**, MDR1: green, indicated by red arrow; nuclei: blue). A reduction in MDR1 expression is further confirmed by immunoblotting of cell lysates from vehicle, Crizo, Dox, or Crizo plus Dox treated HCC cells. Only Crizo plus Dox treatment significantly reduces MDR1 protein level ranging from 40% to 90% in a cell line dependent manner (**Figure 3D**). A summary of results from three independent experiments is shown in **Figure 3E**. Collectively, these results provide evidence that the synergistic effect of Crizo plus Dox in inducing HCC cell death is due to a reduction in the level of MDR1, causing the accumulation of Dox in the nucleus, perpetuating the down-stream responses.

### Crizo Plus Dox Reduce MDR1 Expression by Modulating the Translational Machinery

Next, we investigated the underlying mechanisms by which Crizo plus Dox decrease MDR1 expression. First, we quantified the mRNA levels of MDR1 with three different pairs of primers targeting MDR1 mRNA from 5’- to 3’- (**Supplemental Figure 1**, top panel indicates the positions of the primer pairs) in two HCC cell lines treated with vehicle, Crizo, Dox, or Crizo plus Dox. Cells treated with Dox alone show significant increase in the MDR1 mRNA as detected by all the primer pairs in HepG2 cells. This effect is not detected in cells treated with Crizo alone. On the other hand, the synergist effect of Crizo plus Dox on the MDR1 mRNA level is less clear. It appears to be primer dependent, as well as cell context dependent (**Supplemental Figure 1**). Since Crizo plus Dox do not significantly reduce MDR1 mRNA level, it is unlikely that the down-regulation of MDR1 protein expression is at the transcription level.

**Figure 4:**
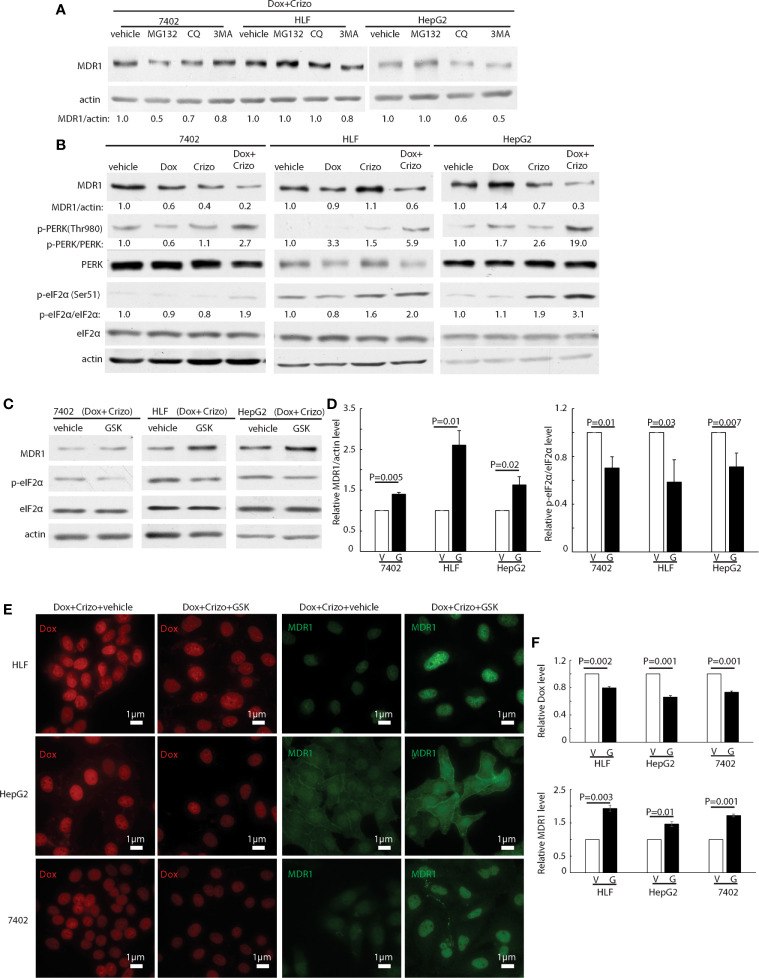
Crizo plus Dox treatment of HCC cells mitigates MDR1 expression by increasing PERK-eIF2α phosphorylation. **(A)** HCC cells treated with Crizo (5 μM) plus Dox (0.85 μM) were incubated with inhibitors for proteasome, autophagy, or lysosome at the same time and cell lysates were blotted with antibodies specific against MDR1 or actin. No significant increase of MDR1 was detected after protein degradation was inhibited by various inhibitors. **(B)** Obviously more phosphorylation of PERK and eIF2α was detected in cells treated with Crizo (5 μM) plus Dox (0.85 μM) than in cells treated with vehicle, Dox (0.85 μM), or Crizo (5 μM) only. Cell lysates from cells under drug treatment for 24 hs were blotted with antibodies specific for indicated proteins, obviously more p-PERK and p-eIF2α signals were observed from cells treated with Crizo plus Dox. **(C)** Significantly more MDR1 was detected from cell lysates after the cells were treated with GSK, a specific inhibitor to PERK, for 2 hs. Effects of GSK on PERK were revealed by immunoblotting against p-eIF2α. Cells were treated with Crizo (5 μM) plus Dox (0.85 μM) for 22 hs and then vehicle or GSK were added for an additional 2 hs before cell lysates were made. **(D)** Open and filled in bar graphs represented the mean and standard deviation from three experiments. **(E)** Immunofluorescence staining for MDR1 showed that 2 hs after GSK addition, obviously more MDR1 was detected in cells treated with Crizo (5 μM) plus Dox (0.85 μM) (the 4^th^ column). In contrast, much less MDR1 was detected in HCC cells treated with vehicle under the same condition (the 3^rd^ column). Obviously more Dox was detected in cells treated with vehicle (the 1^st^ column) whereas much less Dox was observed in cells treated with GSK (the 2^nd^ column). **(F)** Open and filled in graphs represented the mean and standard deviation from three experiments. Relative immunofluorescence intensity or protein levels were determined with IMAGE J as specified. The experiments were repeated at least three times with similar results. V, vehicle; G, GSK. P values were indicated.

We then investigated whether MDR1 protein degradation is enhanced when HCC cells are treated with Crizo plus Dox. For this purpose, in addition to Crizo plus Dox, we also added MG132, a general inhibitor of the proteasome, or chloroquine (CQ), an inhibitor of the lysosome, or 3MA, an inhibitor of autophagy to the cell culture. If reduction of MDR1 expression is due to increases in degradation *via* proteasome or lysosome or autophagy, one or more of these inhibitors should reverse the effects of Crizo plus Dox, with up-regulation of MDR1 level. We find that addition of these inhibitors does not consistently reverse the effects of Crizo plus Dox on MDR1 expression (**Figure 4A**). These results imply that the down regulation of MDR1 protein level is unlikely to be the results of enhanced protein degradation. Thus, MDR1 protein translation may have been impacted by Crizo plus Dox treatment.

Several signaling pathways are involved in protein translation regulation in response to stress (30). To differentiate which pathway is involved in this situation, cell lysates from HCC cells treated with Crizo, Dox or Crizo plus Dox were immunoblotted. It turned out that all three cell lines treated with Crizo plus Dox have obviously higher levels of p-PERK, ranging from 2.7 - 19 times (**Figure 4B**, lane 2). One of the down-stream targets of PERK is (p)-Eukaryotic Initiation Factor 2 alpha (eIF2α). PERK phosphorylates eIF2α to inhibit protein synthesis (31–34). We then investigated whether eIF2α is also altered by Crizo plus Dox. We observe that the levels of p-eIF2α are also significantly up-regulated, across all three treated cell lines, ranging from 1.9- 3.1 times (**Figure 4B**). Up-regulation of eIF2α is known to cause some protein synthesis repression but allows selective translation of activating transcription factor 4 (ATF4) (31–33). As expected, we detected inhibition of protein synthesis in the four HCC cell lines treated with Crizo plus Dox (**Supplemental Figure 2**).

GSK2606414 (GSK) is a specific inhibitor of PERK (35). If our assumption is correct, GSK shall mitigate the effect of Crizo plus Dox. Indeed, when HCC cell lines are cultured with Crizo plus Dox, as well as GSK, the effect of Crizo plus Dox on the down regulation of MDR1 is reversed (**Figure 4C**). Quantification of results from three independent experiments is shown in **Figure 4D**. Accordingly, the levels of p-eIF2α are significantly mitigated (**Figures 4C, D**). Thus, the reduction in MDR1 protein level is likely due to repression of protein translation *via* the PERK-eIF2α signaling cascade.

To seek further support for our interpretation that a reduction in MDR1 expression causes Dox accumulation in the nucleus, we performed immunofluorescence observation for Dox in HCC cell lines treated with Crizo plus Dox. In addition, we also added either GSK or a vehicle as control. As expected, we detect decreased Dox accumulation in the nucleus of cell lines cultured with GSK, with a corresponding increase in MDR1 immunoreactivities (**Figure 4E**). Quantification of results from three independent experiments is shown in **Figure 4F**. Hence, Crizo plus Dox induce endoplasmic reticulum (ER) stress, activates PERK - p-eIF2α signaling pathway, causing a reduction in MDR1 translation, leading to diminished Dox efflux, and accumulation of Dox in the nucleus, resulting in more DNA damages and cell death.

### Crizo Plus Dox Activate Autophagosome Formation *via* PERK-p-eIF2α-JNK Signaling Cascade

**Figure 5:**
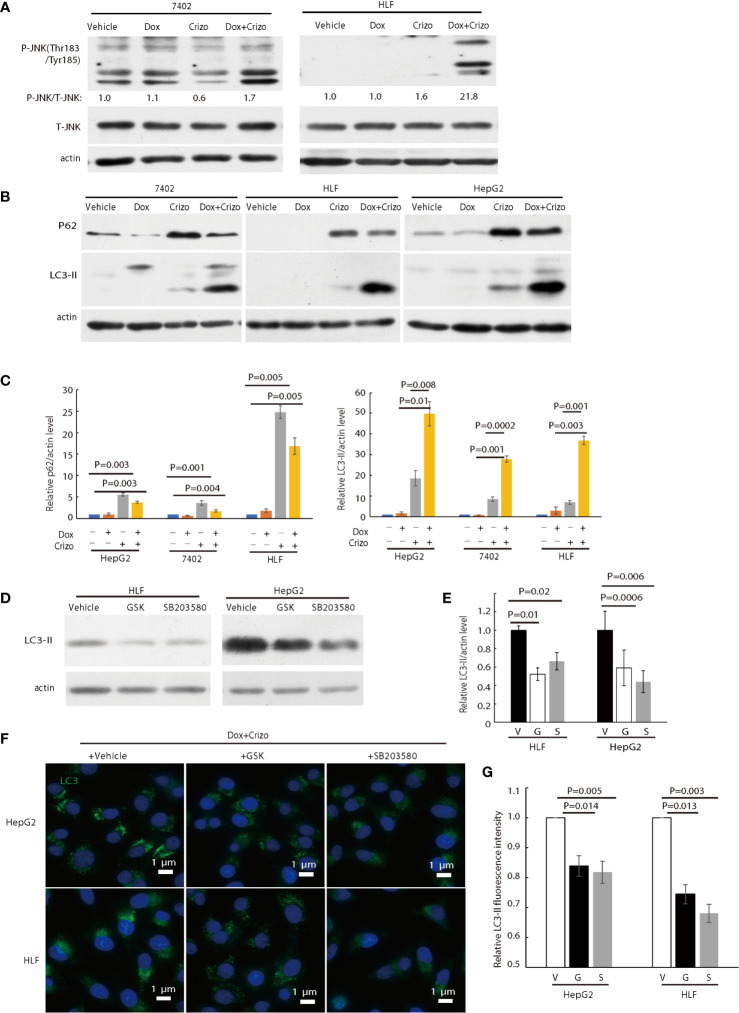
Crizo plus Dox activate PERK-eIF2α-JNK signaling cascade to induce autophagosome formation. **(A)** Cell lysates were blotted with antibodies specific for JNK or phosphorylated JNK, obvious up-regulation of p-JNK was detected in cells treated with Crizo (5 μM) plus Dox (0.85 μM) but not in cells treated with vehicle, Dox (0.85 μM), or Crizo (5 μM) only. **(B)** Significant up-regulation of LC3-II was detected in HCC cells treated with Crizo (5 μM) plus Dox (0.85 μM) compared to that in cells treated with vehicle, Dox (0.85 μM), or Crizo (5 μM) only. Cell lysates were blotted with antibodies specific to p62 or LC3, it turned out more LC3-II was observed when cells were treated with Crizo plus Dox. In addition, p62 was also up-regulated when cells were treated with Crizo plus Dox or with Crizo only. **(C)** Results from four experiments were analyzed. Open and filled in bar graph showed that mean and standard deviation of four experiments. **(D)** Inhibitors for PERK and JNK significantly reduced the formation of LC3-II in cells treated with Crizo (5 μM) plus Dox (0.85 μM) compared to cells treated with vehicle. Immunoblotting was performed with specific antibody against LC3 for cell lysates from cells treated with Crizo plus Dox and vehicle or GSK or SB203580. **(E)** Results from three experiments were analyzed. Open and filled in bar graph showed that mean and standard deviation of three experiments. **(F)** GSK or SB203580 treatment of cells in the presence of Crizo (5 μM) plus Dox (0.85 μM) reduced the formation of autophagosome. Immunofluorescence staining of LC3 in cells treated with Crizo plus Dox and vehicle or GSK or SB203580 showed that inhibition of PERK or JNK activity mitigated the formation of autophagosome. Cells were treated with Crizo plus Dox for 22 hs and vehicle or GSK or SB203580 was added for an additional 2 hs. **(G)** Results from three experiments were analyzed. Open and filled in bar graph showed that mean and standard deviation of three experiments. Fixed cells were stained for LC3 and images were taken after nuclei counter stain. Relative immunofluorescence intensity or protein levels were determined with IMAGE J as specified. V, vehicle; G, GSK; SB, SB203580. P value was indicated.

Since Dox plus Crizo seem to induce ER stress and significantly more cleaved PARP-1 but not more active caspcase-3 (**Figure 2C**), we started to investigate if HCC cells killed by Dox plus Crizo are due to ER stress. c-Jun N-terminal kinase (JNK) is a down-stream of PERK. Activated JNK can markedly induce the formation of autophagosomes (36). Thus, we investigated whether Crizo plus Dox indeed activates JNK. For this purpose, we treated HCC cells with vehicle, Crizo, Dox, or Crizo plus Dox and then immunoblotted the cell lysates. We found obvious increases in the levels of p-JNK in both HCC cell lines treated with Crizo plus Dox (**Figure 5A**), implicating that Dox plus Crizo may activate autophagosome.

LC3-II and p62 are important in autophagosome formation (37, 38). Lipidation of LC3-I generates lapidated LC3-II, allowing the docking of specific cargos, while p62 is a receptor for the cargos destined to be degraded by autophagy (37). Next, we investigated whether Crizo plus Dox modulate the conversion of LC3-I to LC3-II and the expression levels of p62. We find that HCC cell lines treated with Crizo plus Dox indeed have significantly higher levels of LC3-II compared to vehicle, Dox, or Crizo controls across all tested cell lines (**Figure 5B**). Quantifications of results from three independent experiments are shown in **Figure 5C**. On the other hand, the effects of Crizo plus Dox on p62 expression levels are much more intricate (**Figure 5B**). It appears that Dox alone does not affect the expression of p62 noticeably. In contrast, Crizo alone stimulates the expression of p62 in all cell lines as compared to vehicle treated control cells. Unexpectedly, addition of Dox actually mitigates the effects of Crizo a bit (**Figure 5B**). Nonetheless, the effect of Crizo plus Dox is still significantly higher than the vehicle or Dox treated cells. Quantification of results from four independent experiments is shown in **Figure 5C**. Therefore, it is reasonable to assume that Crizo plus Dox also up-regulate the expression of p62.

To confirm autophagosome formation is indeed activated by Crizo plus Dox, we used a well-established protocol of transfecting LC3-GFP into HCC cells and treated the cells with Crizo plus Dox or each drug independently. We observed significantly more autophagosome formation when the transfected HCC cells are treated with Crizo plus Dox (**Supplemental Figure 3A**). Quantification of results from three independent experiments is shown in **Supplemental Figure 3B**. Furthermore, we also performed transmission electron microscopy for cells treated with Dox, vehicle, or Dox plus Crizo. We observe that Dox plus Crizo treated cells have many more vesicles represented by inclusions inside the vesicle (**Supplemental Figure 3C**). Collectively, these results implicate that Dox plus Crizo activate ER stress and enhance autophagosome formation.

To investigate whether the enhanced autophagosome formation is due to PERK-eIF2α-JNK signaling cascade, we also added GSK, or SB203580 (SB), which is a JNK specific inhibitor, to the cell culture in addition to Crizo plus Dox. Both inhibitors significantly reduce the level of the 14 kDa LC3-II protein (**Figure 5D**). Quantification of results from three different experiments is shown in **Figure 5E**. Accordingly, GSK or SB treated HCC cells have significantly lower levels of autophagosome (**Figure 5F**). Quantification of results from three different experiments is shown in **Figure 5G**. To further confirm Dox plus Crizo treatment specifically activate JNK *via* PERK, we silenced PERK with siRNA. Two different siRNAs targeting PERK show efficient down-regulation of PERK; and accordingly, the phosphorylation but not total JNK was impacted (**Supplemental 5**). Thus, Crizo plus Dox activate ER stress and also stimulate autophagosome formation *via* the PERK- eIF2α-JNK signaling cascades.

### Crizo Plus Dox Stimulate Autophagosome and Unfused Autophagosome Contributing to Cell Death

**Figure 6:**
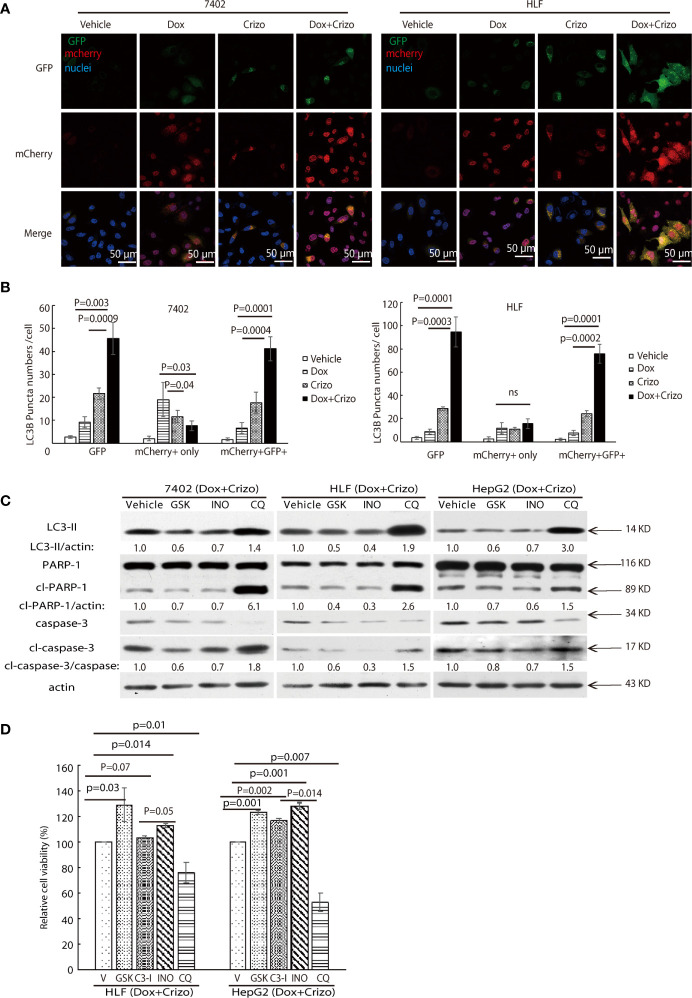
Dox plus Crizo treatment induces PARP-1 dependent cell death. **(A)** Dox plus Crizo induced autophagosome formation but reduced fusion between autophagosome and lysosome. LC3-GFP-mcherry plasmid transfected cells were treated with vehicle, Dox, Crizo, or Dox plus Crizo for 24 hs and subjected for confocal microscopy observation. **(B)** Results from three experiments were analyzed. Open and differentially filled in bar graph showed that mean and standard deviation of three experiments. **(C)** In the presence of Dox plus Crizo, HCC cells were treated with vehicle, GSK, INO, or CQ as indicated. GSK and INO treatment consistently reduced LC3-II, caspase-3 activation (cl-caspase-3), and PARP-1 (cl-PARP-1) cleavage whereas CQ activated LC3-II, cleaved caspase-3, and cleaved PARP-1. The experiments were repeated three times with similar results. **(D)** GSK, INO, and caspase-3 inhibitor (C3-I) treatment decreased Dox plus Crizo’s effects on HCC cells whereas CQ treatment increased such an effect based on MTS assays. Cells seeded as triplicates were treated for 24 hours with vehicle (V), GSK, INO, caspas-3 inhibitor, or CQ in the presence of Dox plus Crizo. The experiments were repeated three times with similar results. P values were indicated. Relative protein levels were determined with IMAGE J as specified. ns, statistically no significant difference.

ER stress has been reported to cause accumulation of p62 resulting in a reduction in autophagosome-lysosome fusion in hepatocytes (39). Accumulation of unfused autophagosome is reported to cause cytotoxicity by promoting cleavage of PARP-1 (27, 40). Besides enhanced levels of LC3-II and autophagosome, Crizo plus Dox treated cells have significantly more p62 (**Figure 5B**). Therefore, we investigate whether Dox plus Crizo treatment have affected fusion between autophagosome and lysosome. We transfected the LC3-GFP-mcherry plasmid into HCC cells and treated those cells with vehicle, Crizo, Dox, or Crizo plus Crizo. We find that cells treated with Crizo plus Dox have significantly more autophagosome than control cells (**Figure 6A**). In addition, cells treated with Dox plus Crizo have significantly higher number of unfused autophagosome than cells treated only with Crizo (**Figure 6A**, unfused autophagosome is indicated as yellow dots; autolysosome is indicated as red dots). Quantification of results from three different experiments is shown in **Figure 6B**. Therefore, Crizo plus Dox indeed activate the formation of autophagosome but decrease the fusion between autophagosome and lysosome.

We then investigated whether the synergistic effects of Dox plus Crizo in killing HCC cells is depended upon PARP-1 cleavage. For this purpose, the following inhibitors: a vehicle control; or GSK; or a PARP-1 inhibitor, INO; or Chloroquine (CQ), a lysosome inhibitor, was added to the cell culture in addition to Crizo plus Dox. After immunoblotting the cell lysates, we find that GSK and INO consistently mitigate the levels of LC3-II (**Figure 6C**, lane 1). In addition, the treatment also resulted in reduced levels of cleaved caspase-3 and cleaved PARP-1 (**Figure 6C**, lanes 2&3&4). Thus, the synergistic effects of Dox plus Crizo on HCC cells are due to mitigated MDR1 translation and are PARP-1 cleavage dependent. It is also likely that PARP-1 cleavage may be a result of enhanced autophagosome when the cells were further treated with CQ. Because, when CQ was added to the Crizo plus Dox cell culture, this treatment did not alter MDR1 level (**Figure 4A**) but greatly enhanced the levels of LC3-II, cleaved caspase-3 and cleaved PARP-1 (**Figure 6C**).

To assess the roles caspase-3, PARP-1, PERK, and autophagosome play in HCC cells treated with Dox plus Crizo, we further treated HCC cells with inhibitor for PERK, caspase-3 (C3-I), PARP-1, or lysosome respectively in the presence of Dox plus Crizo, we observe significantly more cell death when cells are co-treated with CQ but significantly reduced cell death when cells are co-treated with GSK, C3-I, or INO-1001 (INO) (**Figure 6D**), supporting that PERK, caspase-3, PARP-1 cleavage all contribute to cell death caused by Dox plus Crizo. Furthermore, treatment with GSK or INO rescued significantly more HCC cells than treatment with C3-I, suggesting that unfused autophagosome may play a more significant role in inducing HCC cell death through PARP-1 cleavage, a conclusion consistent with our earlier observation (**Figure 2C**).

Next, we investigate whether the synergistic effect of Crizo plus Dox is due to the effect of Crizo on C-Met and ALK; two of the best characterized targets of Crizo (41). It turns out that the effects of Crizo, Dox or Crizo plus Dox vary greatly among the three tested cell lines (**Supplemental Figure S4**). In HepG2 cells treated with Crizo plus Dox, indeed the level of p-MET levels was greatly decreased. This effect is marginal at best in treated Sk-hep1 cells (**Supplemental Figure S4**). In contrast, marginal reduction was observed in HLF cells treated with Dox, or Crizo, or Dox plus Crizo (**Supplemental Figure S4**). We do not detect obvious ALK expression in all the tested HCC cell lines (Results not shown). Hence, synergistic effect by Crizo plus Dox on HCC cells is not depending on its function in inhibiting ALK or/and C-Met activity.

### Crizo Plus Dox Significantly Inhibit Tumor Growth in Vivo in Xenografts

**Figure 7:**
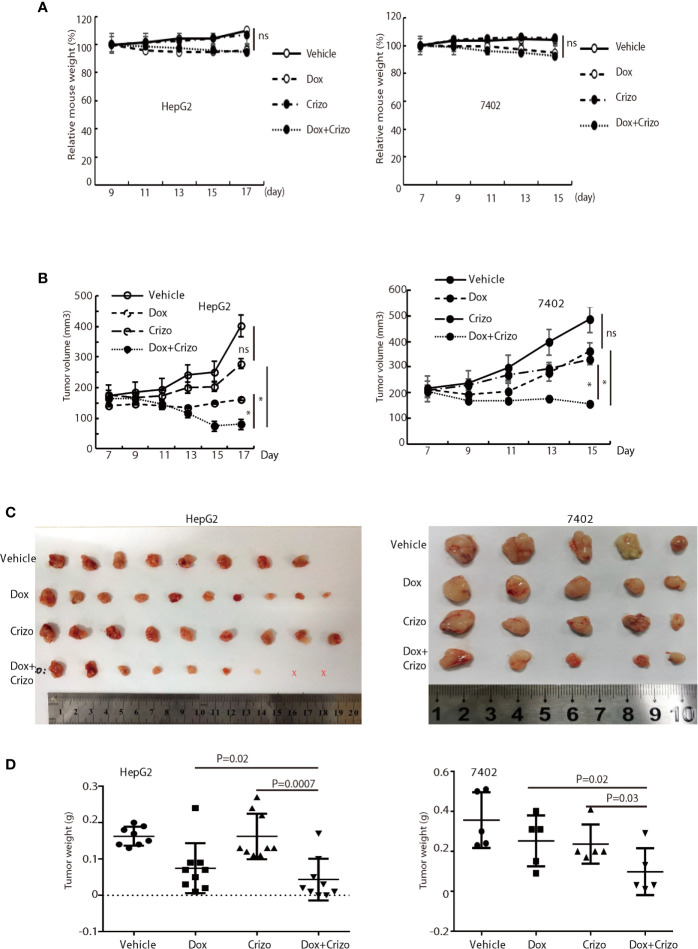
Crizo plus Dox significantly inhibit tumor growth *in vivo* in xenografts. **(A)** Dox, or Dox plus Crizo but not Crizo slightly affected mouse weights. The weight of mouse was monitored every other day as indicated. **(B)** Dox plus Crizo treatment significantly reduced the xenografted tumor volume compared to Dox or Crizo treatment separately. Tumor volumes were measured starting from day 7th post xenograft as indicated. **(C**, **D)** Dox plus Crizo treatment significantly decreased the xenografted tumor weight compared to Dox or Crizo treatment separately. The dissected tumors under different treatments were placed side by side with a scale. Each dot represented the weight of a xenografted tumor. Ns, not statistically significant; *P < 0.05; or P values were indicated. X, tumors disappeared after treatment.

Finally, we investigate where our observations in cell models are applicable *in vivo* in xenograft models. For this purpose, we xenografted HepG2 or 7402 cells subcutaneously in nude mice (BALB/c). Seven days later, all inoculated mice were treated with vehicle, Dox, or Crizo, or Crizo plus Dox. We also appraise the general well-being of all mice treated by monitoring their body weights (**Figure 7A**). It is clear that Crizo plus Dox show synergistic effect in inhibiting tumor growth *in vivo* as monitored by measuring tumor volumes (**Figures 7B**). At the end of the experiment, the tumors were also surgically removed and weighed (**Figure 7C**), and quantified (**Figure 7D**). Indeed, the average tumor weight treated by Dox plus Crizo is decreased by 30% or 65% compared to that treated with Dox or Crizo only for HepG2 cells or by 55% compared to that treated with Dox or Crizo only for 7402 cells, thus, Crizo and Dox show significantly enhanced effect in inhibiting tumor growth *in vitro* as well as *in vivo*.

## Discussion

**Figure 8:**
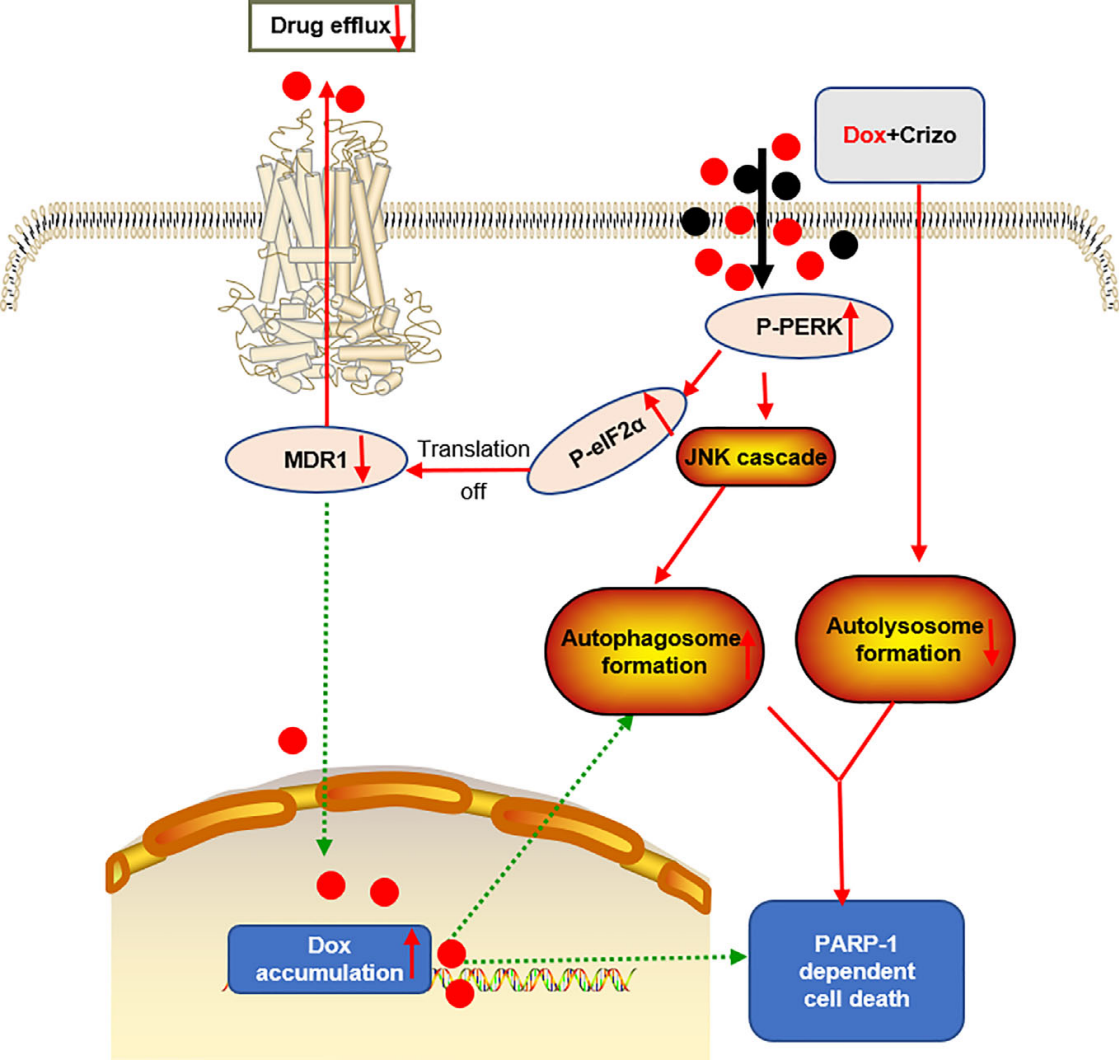
A proposed model to show how Dox plus Crizo act synergistically in killing HCC cells. Crizo plus Dox actiate PERK, which in turn phosphorylates eIF2α, leading to protein translation repression. As a consequence, MDR1 protein level Is reduced, resulting in nuclei accumulation of Dox and DNA damage. In addition, activation of PERK also phosphorylates JNK, leading to autophagosome formation. Somehow, Crizo plus Dox also suppress the fusion between autophagosome and lysosome. All of the mentioned effects cause PARP-1 dependent HCC cell death.

Dox resistance, as well as cardiotoxicity induced by Dox, are the top challenges to treat HCC. To identify TKIs that can effectively enhance Dox’s efficacy, we screen a panel of TKIs and find that Crizo plus Dox act synergistically in inducing cell death *in vitro* using multiple HCC cell lines. More importantly, we unravel the underlying mechanisms by which this synergistic effect arises. Crizo plus Dox treatment enhances cell death by modulating the translational machinery engaging the autophagy pathway. A drawing diagram depicting this process is presented in **Figure 8**.

It is interesting to note that the cell death induced by Crizo plus Dox is further enhanced with the addition of CQ, an observation consistent with other reports (42). More importantly, it has been reported that CQ may improve mid-term survival of patients with glioblastoma multiforme when given in addition to conventional therapy (43). Right now, we are testing if Dox plus Crizo and CQ will further enhance the effect of Dox *in vivo*. While addition of CQ greatly increases LC3-II levels, the underline mechanism how inhibition of lysosomal functions enhanced LC3-II levels and cell death induced by Crizo plus Dox is not known.

To our knowledge, the synergistic effects of Dox and Crizo in inducing HCC cell death have not been reported before. In addition, Dox plus Crizo also show synergism in melanoma cell lines M2 and A375-MA2, in lung cancer cell lines A549 and H460, and in oral squamous cell carcinoma cell line cal27 (data not shown). Hence, Dox plus Crizo may form the foundation in treating different malignant cancers showing MDR1 related drug resistance. While combination regimens of Dox, cisplatin, interferon, or fluorouracil have been explored to treat HCC over the years, the results have been disappointing (14). Our finding that Crizo plus Dox synergistically induce HCC and other cancer cell death is consistent with earlier reports indicating that Crizo acts synergistically with topotecan in killing neuroblastoma cells (44), or combination of Crizo and IGF-1R inhibitor is synergistically cytotoxic to lung cancer cells (45). Dox is known to induce autophagic death in 3T3 cells without involving caspases (27); and activation of PARP-1 has been reported to be involved in apoptotic cell death (46). In this regard, we do observe higher levels of cleaved caspase-3 in HCC cells treated with Crizo or Dox alone in two cell lines, but the synergistic effect of Crizo plus Dox is not seen (**Figure 2C**). Therefore, while apoptosis may contribute to the HCC cell death the role it plays is less pronounced.

We suggest that the synergistic effect of Crizo plus Dox is due to a malicious cycle of signaling cascade with PERK-MDR1 playing a primordial role in this process (**Figure 8**). Since MDR1 modulates the distribution and availability of many drugs, it plays pivotal roles in determining cancer cell susceptibility to many chemotherapeutic drugs (29). The observation that only Crizo plus Dox but neither Dox nor Crizo alone significantly reduced the MDR1 level underpinned the importance using these two drugs together. While it is clear that more Dox in the nucleus will cause more DNA damages, the underlying mechanisms by which Dox plus Crizo caused ER stress remain to be determined. In addition, PERK is one of the three major ER stress sensors (47). Whether the other two sensors, IRE1 and/or ATF6 is/are involved in this process is not known. Another issue we will address in the future is whether eIF2α is the only target of PERK. It is known that PERK also targets Nrf2, FOXO and DAG (47). These signaling transducing intermediates also play critical roles in many biological responses including autophagy; therefore, disrupting one of more of these pathways may further disrupt normal cell physiology. In contrast to our findings Crizo by itself has been reported to inhibit MDR1’s function but not its expression in some human cancer cell lines (48). The reason for this discrepancy is not known at this time. However, there are a few differences between our study and this earlier report. For example, we use different cell lines, as well as different experimental protocols, such as concentrations, and incubation time of the drugs.

Treatment with Dox and Crizo significantly alters the autophagy pathway in HCC cell lines. This finding is not unexpected, because ER stress and autophagy is tightly linked (49, 50). PERK, a target of Crizo plus Dox is involved in both modulating translation machinery and autophagy (33, 51). While it is clear that the pivotal contribution of Dox to the synergistic effect is its accumulation in the nucleus, the role Crizo plays is less clear. Others have reported that treatments with Crizo alone alter autophagy in cancer cell lines (52, 53). In contrast, we find that treating HCC cells with Crizo alone does not significantly activate autophagy, it requires both Dox and Crizo (**Figures 6A, B**). The reason for this discrepancy is not known, and likely to be cell context as well as experimental protocol dependent.

ER stress has been reported to disrupt lysosome homeostasis impairing autophagosome-lysosome fusion (54). Since the autophagosome/lysosome fusion process and lysosome biogenesis are extremely complicated and tightly regulated, elucidating the underlying mechanism for this phenomenon is beyond the scope of this study.

Much higher concentrations of Dox alone have been reported to induce autophagosome formation in cancer cell lines (27, 55). Since we use a much lower concentration of Dox and the effect on autophagosome formation is different when the cells are treated with Dox only, the relevance of these findings to our results is not clear at this time.

The known targets for Crizo are C-Met and ALK. However, Crizo is likely to have off-target effects. Indeed, there are a few studies indicating that Crizo have other targets in addition to ALK and C-Met. For example, reactive oxygen species in NSCLC (56), Akt in gastric cancer (57), and FAK1 in Schwanoma (58). In our study, the effects of Crizo, Dox, or Crizo plus Dox on activation of c-Met are clearly variable, and cell line dependent. On the other hand, ALK is undetectable in the HCC cell lines we tested. Therefore, it is highly unlikely that the synergistic lethality we are reporting here is due to the effects of Crizo on C-Met or ALK.

Although we provide *in vivo* results showing that treatment of mice with Dox plus Crizo does not adversely affect the general well-being of the mice, a much more detailed metabolic study with large group of animals will be needed to consolidate this observation.

In summary, Since Dox and Crizo have already been used extensive in clinics for treating a number of malignancies, our findings reveal a previously unrecognized therapeutic opportunity to control HCC growth in patients with HCC.

## Data Availability Statement

The original contributions presented in the study are included in the article/**Supplementary Material**. Further inquiries can be directed to the corresponding author.

## Ethics Statement

The animal study was reviewed and approved by the animal care and use committee of Wuhan Institute of Virology, Chinese Academy of Sciences.

## Author Contributions

MS and CL designed the experiments. MS performed the experiments. MS, JZ, S-ZC, W-MH, T-YC, G-RW, RS, S-SG, Z-XG, JX, M-SS, and CL analyzed the data. MS and CL wrote the paper. M-SS and CL edited the manuscript. All authors contributed to the article and approved the submitted version.

## Funding

This work was supported by grants from National Science Foundation of China (30670170, 32070147 to CL, 81560442 to JX), from MOST (2018YFA0507201 to CL), from Natural Science Foundation of Guangdong Province (2017ZC0236 to S-ZC), and from Guangzhou Key Medical Discipline Construction Project.

## Conflict of Interest

The authors declare that the research was conducted in the absence of any commercial or financial relationships that could be construed as a potential conflict of interest.

## Abbreviations

Dox, doxorubicin; Crizo, crizotinib; MDR1, multiple drug resistance 1; HCC, hepatocellular carcinoma; PERK, protein kinase RNA-like endoplasmic reticulum kinase; JNK, C-Jun terminal kinase; PARP, Poly-ADP-ribosyltransferase; eIF2α, eukaryotic initiation factor 2.
