# Supplementary material for: Crizotinib and Doxorubicin Cooperatively Reduces Drug Resistance by Mitigating MDR1 to Increase Hepatocellular Carcinoma Cells Death
Source: Front Oncol. 2021 May 18;11:650052. doi: 10.3389/fonc.2021.650052 (PMC8170002; doi:10.3389/fonc.2021.650052)
Supplement: Supplementary file 1 [file DataSheet_1.docx]

Supplemental materials and methods

S1 Quantitative real-time polymerase chain reaction (Q-PCR) assays for MDR1.

After treatment with 0.85 μM Dox or/and 5 μM Crizo for 24 hs, total RNA was extracted using Total RNA Kit I (OMEGA, USA) according to the manufacturer’s instructions. In brief, first-strand cDNA was synthesized from 1 μg of total RNA in a 20 μl total volume by using the PrimeScript 25 °C reagent kit (DRR047A, Takara Bio, Japan). Q-PCR was performed by using SYBR Green Supermix (170-8882AP, Bio-Rad, CA, USA) on a Bio-Rad Connect TM real-time PCR instrument (CFX Connect TM Optics Module). Q-PCR was performed using following program: denaturation at 95 °C for 30 s, followed by 40 cycles of denaturation at 95 °C for 5 s and annealing at 55 °C for 15 s, and a final extension at 70℃ for 20 s. The quantitative mRNA level of MDR-1 was normalized to actin and analyzed by the 2^-ΔΔCt^ method. Primers were listed in supplemental table T2.

S2 autophagic flux detection

To assess autophagic flux, GFP-LC3 plasmid (2 μg) was transfected into 7402, HLF and HepG2 cells in a six-well plate. 12 hs after transfection, cells were trypsinzed, resuspended and seeded in glass bottomed confocal dishes for an additional 24 hs. Fresh full medium containing 0.85 μM Dox or/and 5 μM Crizo were applied to the cells for an extra 12 hs before the cells were subjected to fluorescence microscopy observation as before. GFP-LC3 flux is calculated by the Image J software (NIH).

S3 Autophagosome detection by transmission electron microscope (TEM)

To confirm autophagosome formation during drug treatment, treated cells were washed and fixed in 2.5% glutaraldehyde for 4 h at 4 °C and then treated with 1% osmium tetroxide, dehydrated in a series of graded ethanol baths. The samples were then infiltrated and embedded in Epon resin. Ultrathin sections of 80 nM were cut in a Leica microtome (EMUC7, Germany), poststained with uranyl acetate and lead citrate, and examined in a HITACHI H-7650 transmission electron microscope (TEM) (Tecnai G2 20 TWIN, FEI, America) at an accelerating voltage of 200 kV.

S4 ponceau S staining

To assess the effect of Dox plus Crizo on general protein synthesis, equal numbers of cell after different treatment were lysed in cell lysate buffer as above. The lysates from each indicated treatment were subjected to SDS-PAGE and the separated protein was then transferred to nitrocellulose membrane (Merck Millipore, USA). Once finished, NC membrane was stained in ponceau S solution for 10 minutes. The membrane was then rinsed and the picture was photographed at 30 M.

S5 small interference (si) RNA interference

To validate PERK is the kinase upstream of JNK, we used two siRNAs to silence PERK and detect their effects on JNK phosphorylation by immunoblotting. The target sequences were listed as following: 1#-sense: 5`-GCCAUAAUGGACAUAGUGATT-3`, 1#-antisense: 5`-UCACUAUGUCCAUUAUGGCTT-3`; 2#-sense: 5`-CAAUCUUGCAGUAUCCAUATT-3`, 2#-antisense: 5`UAUGGAUACUGCAAGAUUGTT-3`. Negative control siRNA(NC) , NC-sense: 5`-UUCUCCGAACGUGUCACGUTT-3`, NC-antisense: 5`-ACGUGACACGUUCGGAGAATT-3`; And these oligonucleotides were synthesized from Tsingke Biotechnology, Beijing.

Supplemental Figures and figure legends


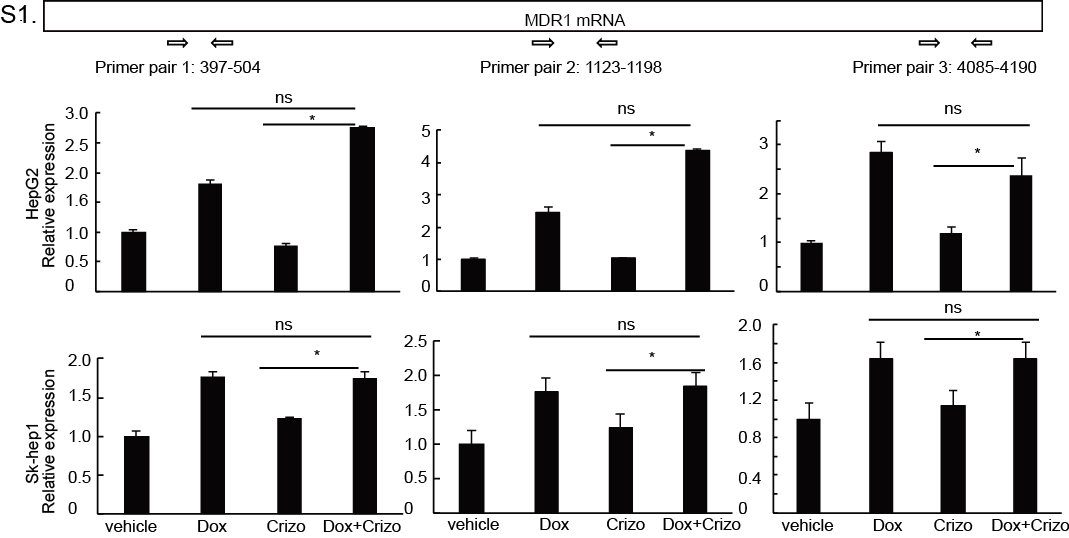


Supplemental figure S1. Quantitative PCR with primers to different parts of MDR1 mRNA showed that Crizo plus Dox treatment did not reduce the amount of MDR1 transcription compared to vehicle, Dox, or Crizo only treatment. Primer pairs targeting sites on MDR1 were indicated and the sequences were listed in supplemental table T2. Total RNA was extracted 24 hs after cell treatment and quantified as described in supplemental materials and methods. These experiments were repeated three times with similar results. ns: not statistically significant; *: P<0.05.


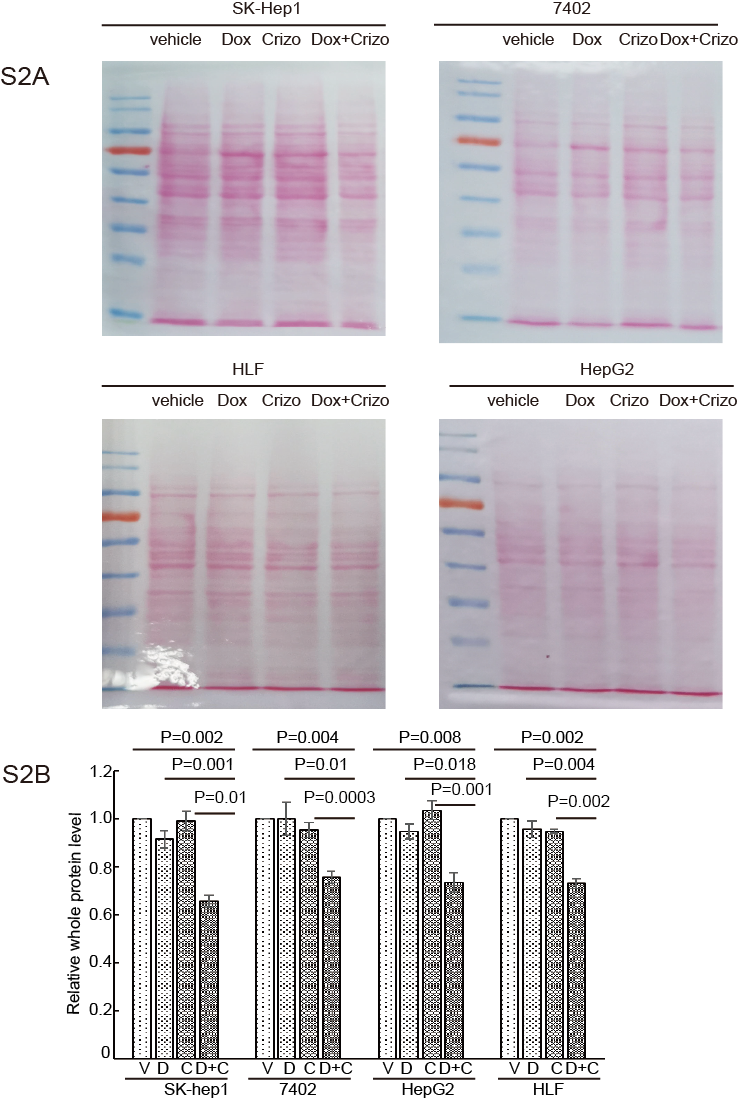


Supplemental figure S2. (S2A) Ponceau S staining of total cell lysates of HCC cells treated with vehicle, Dox, Crizo, or Dox plus Crizo showed that general protein synthesis was significantly suppressed when cells were treated with Dox plus Crizo. The same number of cells was counted and cell lysates were made and subjected to 10 % SDS-PAGE. After gel electrophoresis, the separated proteins were stained with ponceau S as described in supplemental materials and methods. These experiments were repeated three times with similar results. (S2B) A summary of three experiments showed as differentiate filled in bar graph. Mean and standard deviation were from three independent experiments. P values were indicated.


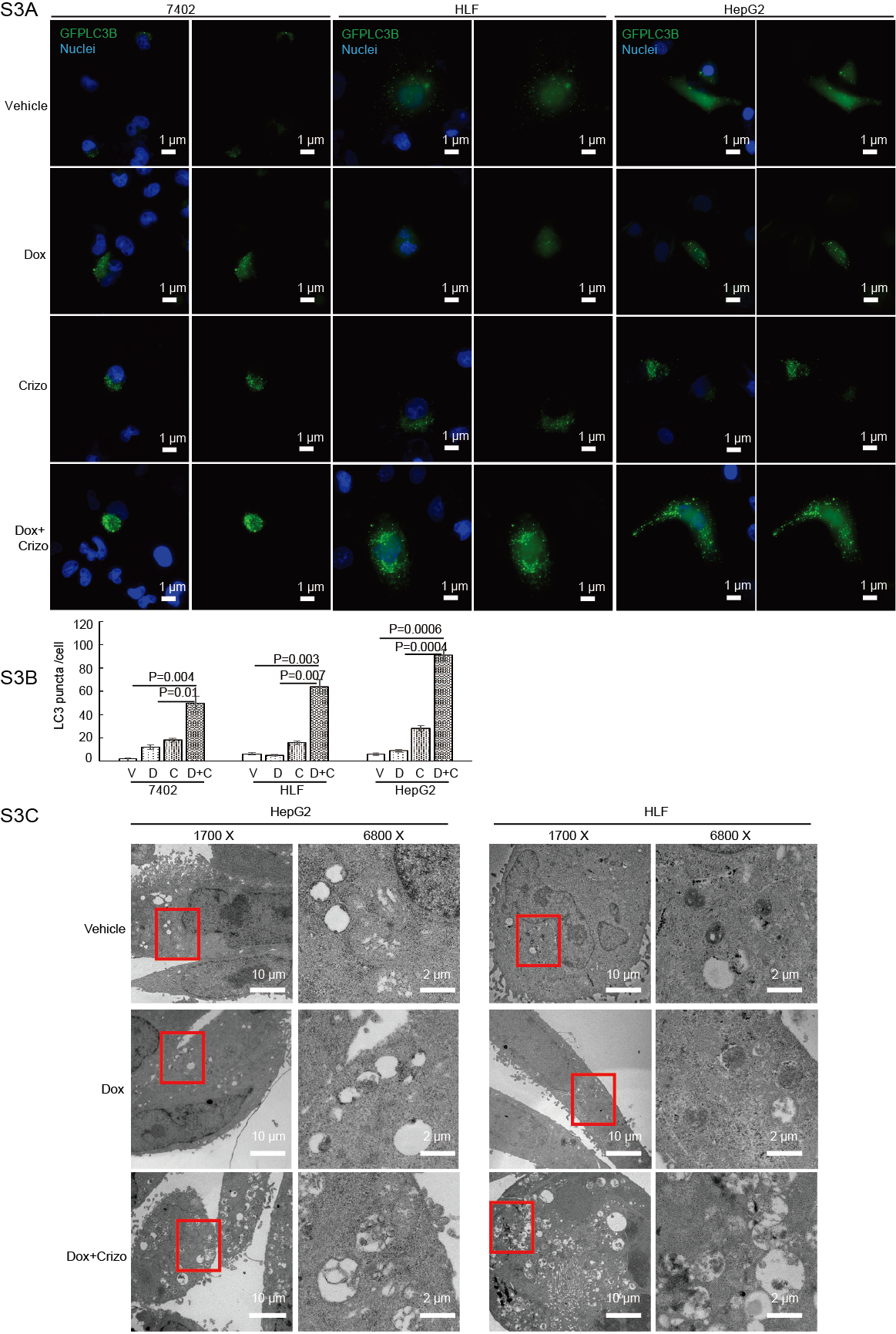


Supplemental figure S3. (S3A) Dox plus Crizo treatment induced much more autophagosome than vehicle, Dox, or Crizo treatment only. LC3-GFP transfected cells were treated as indicated and cultured for 24 hs. The cells were then fixed and subjected to confocal immunofluorescence microscopy for imaging. (S3B) A summary of three experiments showed as differentiate filled in bar graph. Mean and standard deviation were from three independent experiments. P values were indicated. (S3C) Transmission electron microscopy showed that Dox plus Crizo treated HCC cells had much more vesicles with inclusion inside than Dox or Vehicle treated cells. Transmission electron microscopy was performed as described in supplemental materials and methods. The images were taken with Transmission electron microscopy (Tecnai G­2 20 TWIN, FEI, America). These experiments were repeated three times with similar results. V: vehicle; D: Dox; C: Crizo; D+C: Dox plus Crizo.


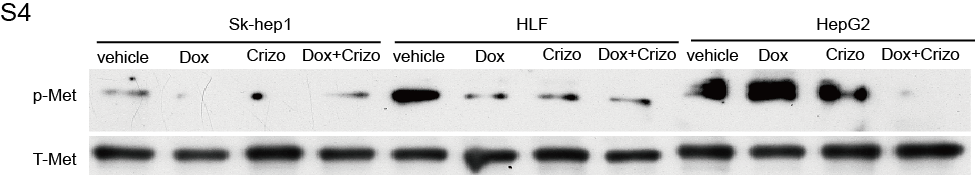


Supplemental figure S4. Dox plus Crizo treatment did not consistently reduce C-Met activity compared to Dox or Crizo treatment only. HCC cells were cultured for 24 hs and cell lysates were made. C-Met (cell signaling technologies) or p-Met (cell signaling technologies) was detected with polyclonal antibodies specific to corresponding protein. All cells express Met. However, HLF and HepG2 cells had obvious p-Met level which was reduced greatly by Dox plus Crizo treatment. In contrast, Dox or Crizo only also reduced p-Met level in HLF cells. These experiments were repeated three times with similar results.


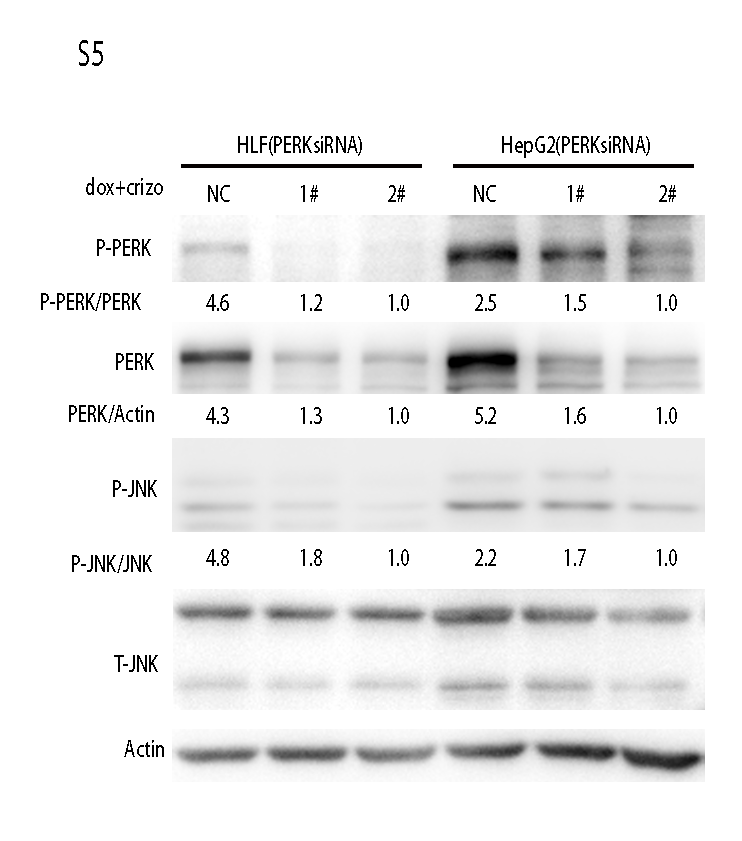


Supplemental figure S5. PERK knock-down significantly inhibits JNK phosphorylation under dox plus crizo treatment. HLF and HepG2 cells were cultured for 24 hours, then transiently transfected with control and PERK siRNAs targeting two different sites, respectively. 24 hours post transfection, cells were cultured with dox plus crizo for additional 24 hours before cell lysates were made. The target proteins were detected by western blot. The PERK siRNAs efficiently down regulate PERK; and phosphorylated JNK levels were repressed accordingly. NC: negative control.

Supplemental Table T1. List of concentration combinations used in determining CI values.

| Cell line | Dox (μM) | Crizo (μM) | Fa | CI value |
| --- | --- | --- | --- | --- |
| 7402  SK-hep1  HLF  HepG2 | 0.6  0.6  0.85  0.85  0.85  0.85  0.85  1.28  1.28  1.28  1.28  0.6  0.6  0.85  0.85  0.85  0.6  0.85  0.85  0.6  0.34  0.34  0.6  0.6  0.85  0.85  1.28  1.28  1.28  0.85  0.85  0.85  0.6  0.34 | 1.0  2.5  1.0  2.5  5.0  7.5  10.0  1.0  2.5  5.0  10.0  10.0  2.5  2.5  5.0  7.5  7.5  7.5  10.0  10.0  7.5  10.0  2.5  5.0  2.5  0.5  2.5  5.0  7.5  5.0  7.5  10.0  10.0  10.0 | 0.34  0.43  0.46  0.54  0.78  0.88  0.96  0.72  0.85  0.89  0.95  0.97  0.82  0.84  0.91  0.93  0.91  0.91  0.96  0.97  0.65  0.98  0.68  0.87  0.84  0.86  0.84  0.84  0.86  0.69  0.76  0.93  0.93  0.91 | 0.875  0.924  0.766  0.813  0.586  0.523  0.35  0.428  0.328  0.395  0.415  0.583  0.797  0.949  0.879  0.922  0.239  0.263  0.189  0.144  0.55  0.117  0.341  0.231  0.229  0.145  0.303  0.385  0.423  0.775  0.942  0.63  0.64  0.70 |

Supplemental Table T2. The primers used for PCR amplification

| primers | sequences |
| --- | --- |
| Actin-F  Actin-R  MDR1-F1  MDR1-R1  MDR1-F2  MDR1-R2  MDR1-F3  MDR1-R3 | 5’-agaaaatctggcaccacacc-3’  5’-tgatctgggtcatcttctcg-3’  5’-ccgctgttcgtttcctttag-3’  5’-tcaagatccattccgacctc-3’  5’-tggttggaagctaacccttg-3’  5’-tatctttgcccagacagcag-3’  5’-cttttggatgaagccacgtc-3’  5’-ggtgagcaatcacaatgcag-3’ |
